# Evaluation der Durchgangsarztberichte in der Deutschen Gesetzlichen Unfallversicherung – Methodik und Ergebnisse eines Peer-Review-Verfahrens

**DOI:** 10.1007/s00113-020-00824-4

**Published:** 2020-06-02

**Authors:** D. Szczotkowski, C. Neik, U. Polak, M. Wittwer, T. Kohlmann

**Affiliations:** 1 Institut für Community Medicine, Universitätsmedizin Greifswald, Walther-Rathenau-Str. 48, 17475 Greifswald, Deutschland; 2 Deutsche Gesetzliche Unfallversicherung e. V., Glinkastraße 40, Berlin, 10117 Deutschland

**Keywords:** Checkliste, Arztberichte, Arbeitsunfall, Qualitätssicherung, Prozessqualität, Checklist, Occupational accident, Medical reports, Quality assurance, Process quality

## Abstract

**Hintergrund:**

Durch eine bundesweite Beurteilung von Durchgangsarzt(D)-Berichten sollte die Dokumentationsqualität im Durchgangsarztverfahren der Deutschen Gesetzlichen Unfallversicherung (DGUV) systematisch erfasst werden. Peer-Review-Verfahren sind bewährte Instrumente zur Sicherung der Prozessqualität.

**Material und Methoden:**

Für eingeschlossene D‑Ärzte wurden zufällig 30 D-Berichte mit besonderer Heilbehandlung aus dem Jahr 2017 ausgewählt, anonymisiert und webbasiert von einem zufällig zugeteilten Beurteiler (Peer) mit einer Checkliste bewertet. Diese umfasste 9 Beurteilungskategorien mit dichotomem Antwortformat (Mangel/kein Mangel). Zur Gesamteinschätzung wurde für jeden D‑Bericht eine Gesamtnote („sehr gut“ bis „ungenügend“) vergeben.

**Ergebnisse:**

Insgesamt bewerteten 82 Peers 30.384 D‑Berichte. Jeder dritte D‑Bericht wies keine Mängel auf. Die häufigsten Mängel waren in der Beurteilungskategorie „Angaben zum Unfall“ zu beobachten. Die durchschnittliche Gesamtnote betrug 2,6, wobei es auf der Ebene der evaluierten D‑Ärzte deutliche Qualitätsunterschiede gab (beste: 1,5; schlechteste: 4,1). Alle evaluierten D‑Ärzte erhielten individuelle Qualitätsberichte, in denen die Kernergebnisse beschrieben wurden.

**Schlussfolgerung:**

Das erste bundesweite Peer-Review der DGUV konnte sich als praktikables und valides Qualitätssicherungsverfahren für D‑Berichte bewähren. Die Qualität der Berichte kann allgemein als gut eingeschätzt werden. Die DGUV plant, das Verfahren unter Berücksichtigung weiterer D‑Arzt-Gruppen zu wiederholen.

## Abstract (en)

**Background:**

Accident insurance consultants (D-physicians) are qualified specialists with particular expertise in occupational medicine. Within the medical treatment procedure of the German Statutory Accident Insurance (DGUV), D‑physicians must make a report on the medical care after occupational accidents. This nationwide evaluation aimed to systematically measure the quality of documentation of these medical reports. Peer review is a common method to ensure process quality.

**Material and methods:**

For each included D‑physician 30 reports of more severe cases from 2017 were randomly selected. The reports were anonymized and randomly assigned to a peer reviewer. Peer reviewers used a web-based checklist with nine rating categories and dichotomous response format (deficiency/no deficiency). To evaluate overall quality each report was rated with an overall grade from 1 (very good) to 6 (insufficient).

**Results:**

A total of 30,384 reports were evaluated by 82 peer reviewers. One third of the reports contained no deficiencies. Most deficiencies were found in the category on information about the accident. The mean overall grade for each D‑physician was 2.6 and ranged from 1.5 (best) to 4.1 (worst). All evaluated D‑physicians were given an individual quality report which described the main findings.

**Conclusion:**

The first nationwide peer review of the DGUV proved to be a practical and valid quality assurance procedure to evaluate the medical reports of D‑physicians. The quality of the reports was in general good. The DGUV plans to repeat the peer review process taking further groups of D‑physicians into consideration.

Die Deutsche Gesetzliche Unfallversicherung e. V. (DGUV) ist der Spitzenverband der gewerblichen Berufsgenossenschaften und der Unfallversicherungsträger der öffentlichen Hand (UV-Träger). Ihr satzungsgemäßer Auftrag ist es, die Struktur‑, Prozess- und Ergebnisqualität von Rehabilitationsmaßnahmen und Maßnahmen zur Teilhabe zu sichern [4]. Dies schließt das ambulante und stationäre Heilverfahren ein. Das Peer-Review für Durchgangsarzt(D)-Berichte stellt vor diesem Hintergrund eine Maßnahme zur Sicherung der Prozessqualität im Durchgangsarztverfahren dar.

## Hintergrund und Fragestellung

Mit der Neuausrichtung ihrer Heilverfahren begannen auch die Planungen der DGUV für eine systematische Evaluation der Durchgangsarzt(D)-Berichte in Form eines Peer-Reviews [22]. Dieses international etablierte Verfahren ist ganz allgemein dadurch gekennzeichnet, dass unabhängige Fachkollegen^1^ eine Qualitätseinschätzung anhand standardisierter Kriterien vornehmen (englisch „Peers“ für Ebenbürtige; „Review“ für Überprüfung) [9, 13]. Hinsichtlich der praktischen Ausgestaltung existieren jedoch selbst innerhalb des deutschen Gesundheitswesens unterschiedliche Herangehensweisen. So setzt die Bundesärztekammer seit 2009 mit dem Curriculum „Ärztliches Peer Review“ auf eine Kombination aus Selbst- und Fremdbewertung, bei der die Peers im Rahmen von Visitationen durch Interviews und Beobachtungen ihre Erkenntnisse gewinnen und noch vor Ort mit den zu bewertenden Einrichtungen diskutieren [2, 3]. Eine andere Möglichkeit stellt die Qualitätseinschätzung auf der Grundlage von medizinischen Aufzeichnungen dar, wie es im seit nunmehr 20 Jahren bewährten Peer-Review-Verfahren der gesetzlichen Rentenversicherung der Fall ist. Bis zu 20 anonymisierte Entlassungsberichte pro Rehabilitationseinrichtung werden hier von geschulten Rehabilitationsmedizinern mit einer indikationsspezifischen Checkliste begutachtet [9]. Mit ihrem Peer-Review verfolgt die DGUV einen ganz ähnlichen Ansatz ebenfalls auf Basis eines medizinischen Dokuments.

### Die Bedeutung des Durchgangsarztberichts

Die Erstellung und Übermittlung des D‑Berichts ist der Ausgangspunkt eines jeden Durchgangsarztverfahrens (D-Arzt-Verfahrens) nach einem Arbeitsunfall. Durch diesen Bericht erhält der UV-Träger die relevanten Informationen zur Prüfung des Versicherungsfalls. Der D‑Arzt dokumentiert in dem unverzüglich zu erstattenden D‑Bericht (§ 27 (2) Vertrag Ärzte/Unfallversicherungsträger) nicht nur medizinische Aspekte wie die klinische Befunderhebung, diagnostische Maßnahmen, die Erstdiagnose oder die Erstversorgung. Er hält auch die in der Regel noch unbeeinflusst erteilten Erstangaben des Verletzten zum Unfallhergang fest, denen eine besondere versicherungsrechtliche Bedeutung zukommt (z. B. Hessisches LSG Urteil v. 24.03.2010 – L3 U 225/10) [24]. Nicht zuletzt bestimmt der D‑Arzt mit dem D‑Bericht auch die einzuleitende Art des Heilverfahrens und damit dessen Steuerung. Aufgabe der zuständigen UV-Träger ist es, die Voraussetzungen für eine Leistungsbewilligung zu ihren Lasten zu prüfen. Fehlerhafte oder unvollständige Angaben können diesen Prozess durch zeitaufwendige Rückfragen bei den D‑Ärzten unnötig erschweren, eigentlich berechtigte Ansprüche von Versicherten infrage stellen oder wiederum zur Bewilligung ungerechtfertigter Leistungen führen [19, 23, 26]. Frühere, allerdings stichprobenhafte Überprüfungen lieferten bereits Hinweise auf fehlerhaft ausgefüllte D‑Berichte in der Routinedokumentation [21].

### Vom Forschungsprojekt zum bundesweiten Peer-Review

Dem hier vorgestellten, bundesweiten Peer-Review ging zunächst ein Forschungsprojekt voraus, in dem in Zusammenarbeit mit D‑Ärzten sowie Vertretern der DGUV und der UV-Träger eine Checkliste zur Beurteilung von D‑Berichten erfolgreich erprobt wurde [25]. Die Checkliste war dabei integriert in eine eigens entwickelte Internetanwendung C‑DAB („Checkliste für Durchgangsarztberichte“), sodass die Peers D‑Bericht-Fälle computergestützt bewerteten. Die DGUV beschloss im Rahmen ihrer Qualitätssicherung, die in dem Forschungsprojekt entwickelten Instrumente für eine bundesweite Evaluation von D‑Berichten zu nutzen, und beauftragte neuerlich das Institut für Community Medicine der Universitätsmedizin Greifswald (ICM) mit der Durchführung dieses Vorhabens [19].

Ziel des Projektes war es auch, den evaluierten D‑Ärzten in Form von individuellen Qualitätsberichten und im Sinne eines Benchmarkings eine Rückmeldung zu ihrer Dokumentationsqualität zu geben.

## Methodik

### Die eingesetzte Checkliste als Erhebungsinstrument

Die Beurteilung der D‑Berichte erfolgte mit der Checkliste aus dem Forschungsprojekt, welche aufgrund leicht veränderter Rahmenbedingungen geringfügig modifiziert wurde. Dabei wurde die Inhaltsvalidität der Formulierungen von dem interdisziplinären Projektteam (4 D‑Ärzte, 6 UV- und 2 ICM-Mitarbeiter) nochmals überprüft. Die final eingesetzte Checkliste umfasste 9 Beurteilungskategorien mit jeweils einer Frage (Tab. 1). Das Vorliegen eines Mangels war durch die Beantwortung der jeweiligen Checklistenfrage entweder zu verneinen oder zu bejahen. Hierbei stellten die Vollständigkeit und Schlüssigkeit der D‑Bericht-Angaben die wichtigsten übergeordneten Kriterien dar. Wurde ein Mangel festgestellt, so ließ sich die Relevanz dieses Mangels auf einer Skala von 1 bis 10 einschätzen (1: sehr geringe, 10: sehr starke Beeinträchtigung der Berichtsqualität durch den Mangel). Zusätzlich gab es die Möglichkeit, den Mangel durch einen Freitextkommentar näher zu beschreiben. Abschließend war die allgemeine Berichtsqualität mit einer Gesamtnote zu bewerten (1: sehr gut, 6: ungenügend).

**Table 1:** 

Beurteilungskategorie	Checklistenfrage (Antwortformat jeweils „ja“/„nein“)
*Angaben zum Unfall*	Sind die Angaben (zu Unfallort, Unfallhergang und zur Tätigkeit der/des Versicherten zum Unfallzeitpunkt) Ihrer Meinung nach vollständig?
*Klinische Befunde*	Sind die für diesen Verletzungsmechanismus relevanten klinischen Befunde und die klinische Funktionsuntersuchung dokumentiert worden?
*Röntgenentscheidung*	Ist die Röntgenentscheidung schlüssig?
*Röntgenergebnis*	Ist das beschriebene Röntgenergebnis ausreichend und vollständig? (*Optional*)^a^
*Erstdiagnose*	Folgt die beschriebene Erstdiagnose schlüssig aus der Beschreibung des Unfallhergangs, der Befunde und ggf. der Röntgendiagnostik?
*Erstversorgung*	Sind die beschriebenen Maßnahmen die schlüssige Folge des Unfallhergangs und des Befunds?
*Annahme Arbeitsunfall*	Spricht etwas gegen die Annahme eines Arbeitsunfalls?
*Art der Heilbehandlung*	Erscheint die Einleitung der besonderen Heilbehandlung schlüssig?
*Klassifikation der Verletzung*	Ist die Klassifikation der Verletzung nach VAV/SAV zutreffend?

*VAV* Verletzungsartenverfahren, *SAV* Schwerstverletzungsartenverfahren [6]

^a^Sofern eine Röntgendiagnostik durchgeführt wurde

### Einschlusskriterien

Beurteilt wurden nur D‑Berichte aus dem Jahr 2017 (auch: DABE/F1000), mit denen eine besondere Heilbehandlung eingeleitet wurde. Dies schloss auch Wege- und Schulunfälle mit ein. Besonderer Heilbehandlung liegen grundsätzlich eher schwerere Verletzungen zugrunde [7]. Es gab 2 Gruppen von zu evaluierenden Leistungserbringern:

- *niedergelassener Bereich*: D‑Arzt-Praxen mit jährlich mindestens 50 Fällen an besonderer Heilbehandlung laut DGUV-Statistik,
- *SAV-Bereich*: Alle D‑Ärzte aus unfallchirurgischen oder kinderchirurgischen Kliniken an Krankenhäusern mit einer Zulassung zum Schwerstverletzungsartenverfahren (SAV).

### Technische Aspekte

Alle eingeschlossenen D‑Berichte lagen als XML-Dateien vor und wurden automatisch durch die IT-Abteilung der DGUV anonymisiert, wodurch keine Rückschlüsse auf die Identität der Versicherten gezogen werden konnten. Als Zuordnungsmerkmal für die Leistungserbringer verblieb lediglich das Institutionskennzeichen (IK) in der XML-Datei. Dies war jedoch nur den zuständigen Mitarbeitern des ICM und nicht den Peers bekannt.

Aus dem gesamten Fallaufkommen von ca. 270.000 D‑Berichten wurde eine Zufallsstichprobe gezogen, bei der als Zielvorgabe aus allen 4 Quartalen 2017 pro Leistungserbringer insgesamt 30 D-Berichte auszuwählen waren. Die ausgewählten D‑Berichte wurden in die Internetanwendung C‑DAB geladen und dort wiederum zufällig einem Peer zugeordnet. Durch das IK konnte technisch gewährleistet werden, dass kein Peer einen seiner eigenen D‑Berichte erhielt.

**Figure 1:**
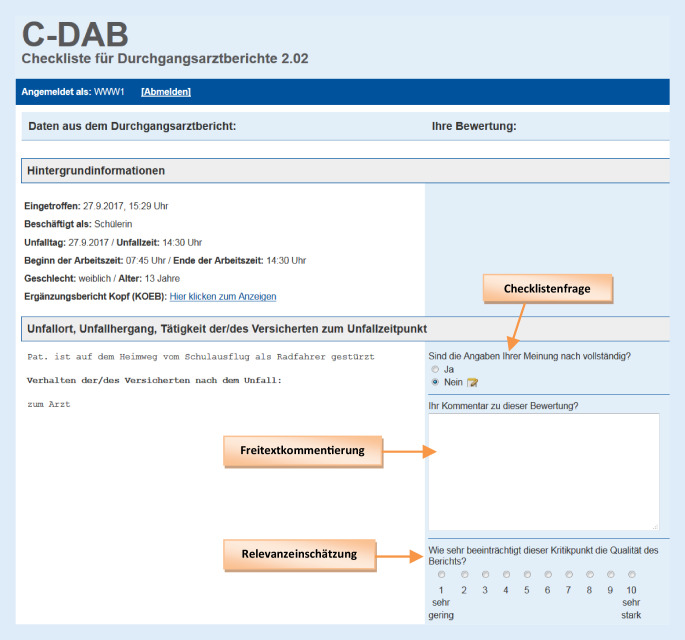

Die Bewertungen fanden im Zeitraum zwischen April 2017 und April 2018 statt. Der Login in C‑DAB erfolgte passwortgeschützt über eine SSL-gesicherte Internetverbindung und war standortunabhängig möglich. Über einen Peer-Account waren pro Woche durchschnittlich 10 bis 20 D-Berichte abrufbar. Die Benutzeroberfläche in C‑DAB war so gestaltet, dass auf der *linken (weißen) Seite* die Originalangaben aus dem D‑Bericht dargestellt wurden, während auf der *rechten (hellblau eingefärbten) Seite* die zugehörigen Checklistenfragen zu beantworten waren (Abb. 1). Neben Hintergrundinformationen wie u. a. dem Geburtsjahr und Geschlecht des Versicherten oder dem Unfallzeitpunkt konnte der Peer auch ggf. anhängige Ergänzungsberichte für Kopf- (KOEB/F1002) und Knieverletzungen (KNEB/F1004) sowie für Verbrennungen (VEEB/F1008) einsehen.

### Peers

Nach bundesweiter Information Ende 2016 über das anstehende Projekt, u. a. mit der Hilfe der unfallchirurgisch-orthopädischen Berufsverbände und Fachgesellschaften sowie der Landesverbände der DGUV, zeigten 270 aktive D‑Ärzte, anerkannte D‑Arzt-Vertreter sowie Beratungsärzte Interesse an einer Tätigkeit als Peer. Durch die geplante Mengensteuerung konnte jedoch nur eine begrenzte Anzahl an Peers berücksichtigt werden, weshalb die Landesverbände der DGUV eine Auswahl treffen mussten. Eine langjährige Erfahrung als D‑Arzt stellte hierbei das wichtigste Kriterium dar. Ambulant tätige Peers erhielten nur D‑Berichte aus dem niedergelassenen Bereich, während Peers mit SAV-Erfahrung nur D‑Berichte aus SAV-Krankenhäusern zugeteilt wurden.

Zur Vorbereitung auf ihre Tätigkeit bekamen die Peers ein Manual mit ausführlichen Erläuterungen und Fallbeispielen zu den Beurteilungskriterien. Die wesentlichen Inhalte des Manuals konnten hierbei in Form von Hilfstexten auch in C‑DAB abgerufen werden. Nach dem ersten erfolgreichen Login in C‑DAB musste jeder Peer überdies zunächst drei fiktive Übungsfälle mit unterschiedlichen Lernschwerpunkten und Hinweisen zur richtigen Bewertung bearbeiten. Erst danach konnten Echtfälle bewertet werden.

Um ein systematisches Feedback zum Verfahren zu erhalten, wurden die Peers nach Ende ihrer Tätigkeit gebeten, an einer anonymen Onlinebefragung teilzunehmen, die mithilfe der Umfragesoftware LimeSurvey erstellt wurde.

### Auswertungen

Deskriptive Auswertungen sowie die Berechnungen linearer und logistischer Regressionsanalysen erfolgten mit SPSS Statistics 25. Freitextnennungen wurden mit MAXQDA qualitativ analysiert und kategorisiert.

Zur Bestimmung der Interrater-Reliabilität wurden vom Projektteam für den niedergelassenen Bereich 5 D‑Berichte und für den SAV-Bereich 10 D‑Berichte ausgewählt, welche allen Peers der jeweiligen Gruppe vorgelegt wurden. Die Peers sind über das Vorhaben informiert worden, jedoch waren die ausgewählten D‑Berichte nicht als solche in C‑DAB gekennzeichnet. Für den in der Literatur selten beschriebenen Fall von mehr als 2 Beurteilern, welche dichotome Beurteilungskriterien mit asymmetrischen Randhäufigkeiten einschätzen, wurde die prozentuale Beurteilerübereinstimmung berechnet. Diese gibt die Wahrscheinlichkeit an, mit der ein zufällig ausgewählter Beurteiler dasselbe Urteil wie ein anderer zufällig ausgewählter Beurteiler fällt [15]. Zur Einschätzung der Beurteilerübereinstimmung hinsichtlich der mindestens ordinalskalierten Gesamtnoten wurde der Finn-Koeffizient bestimmt. Hierzu wird das Verhältnis aus beobachteter und – im Fall zufälliger Notenvergabe – zu erwartender Varianz von 1 subtrahiert [11]. Finn-Koeffizienten zwischen 0,5 und 0,7 können als zufriedenstellend, darüber hinausgehende Werte als gut angesehen werden [9].

## Ergebnisse

Im niedergelassenen Bereich bewerteten 68 Peers insgesamt 27.116 D‑Berichte. Im SAV-Bereich wurden 3268 D‑Berichte von 14 Peers beurteilt. Die Stichprobenmerkmale sind in Tab. 2 dargestellt.

**Table 2:** 

	Niedergelassener Bereich		SAV Bereich		Gesamt		*p*-Wert*
	*n*	(%)	*n*	(%)	*n*	(%)	
*Bewertete D‑Berichte*	*27.116*	*(100)*	*3268*	*(100)*	*30.384*	*(100)*	–
*Alter der verletzten Person*							
in Jahren (MW ± SD)	34,1 ± 18,7		32,8 ± 19,9		34,0 ± 18,9		<0,001^a^
Minderjährig	7816	(28,8)	986	(30,2)	8802	(29,0)	0,109^b^
*Geschlecht der verletzten Person*							
Weiblich	9123	(33,6)	1063	(32,5)	10.186	(33,5)	0,201^b^
Männlich	17.993	(66,4)	2205	(67,5)	20.198	(66,5)	
*Behandlungstyp*							
Ambulant	25.331	(93,4)	1661	(50,8)	26.992	(88,8)	<0,001^b^
Stationär	957	(3,5)	1598	(48,9)	2555	(8,4)	
Keine Angabe im D‑Bericht	828	(3,1)	9	(0,3)	837	(2,8)	–
*Verletzung nach VAV/SAV* [6]							
Nein	26.796	(98,8)	2494	(76,3)	29.290	(96,4)	<0,001^b^
VAV	264	(1,0)	573	(17,5)	837	(2,8)	
SAV	56	(0,2)	201	(6,2)	257	(0,8)	
*Anzahl der Diagnosen*							
MW ± SD	1,5 ± 1,0		1,8 ± 1,6		1,6 ± 1,1		<0,001^a^
*Fehlende Angaben im D‑Bericht bei …*							
der Art der Beschäftigung	1199	(4,4)	145	(4,4)	1344	(4,4)	0,968^b^
den Beschäftigungszeiten	2850	(10,5)	1016	(31,1)	3866	(12,7)	<0,001^b^
der Unfallzeit	489	(1,8)	208	(6,4)	697	(2,3)	<0,001^b^
*Umfang der D‑Berichte* Anzahl der Zeichen (MW ± SD)	631 ± 313		1048 ± 636		676 ± 384		<0,001^a^

*n* Fallzahl, *MW* Mittelwert, *SD* Standardabweichung, *VAV* Verletzungsartenverfahren, *SAV* Schwerstverletzungsartenverfahren

*Test auf Unterschiede zwischen dem niedergelassenen und dem SAV-Bereich: ^a^t‑Test, ^b^Chi-Quadrat-Test

### Mängelfeststellungen

**Figure 2:**
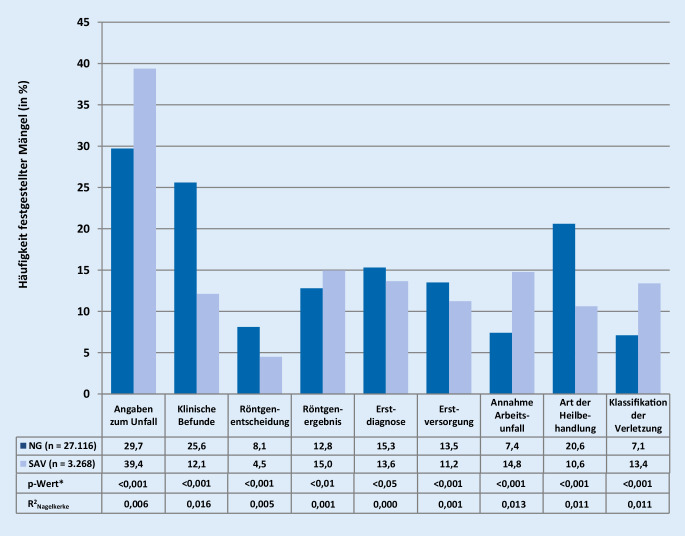

Jeder dritte D‑Bericht war ohne Mängel. Im niedergelassenen Bereich traten die meisten Mängel in den Beurteilungskategorien Angaben zum Unfall, klinische Befunde sowie Art der Heilbehandlung auf (Häufigkeit festgestellter Mängel jeweils über 20 %), während im SAV-Bereich nur die Beurteilungskategorie Angaben zum Unfall mit knapp 40 % festgestellten Mängeln auffällig war (Abb. 2). Zwischen dem niedergelassenen und dem SAV-Bereich waren zwar einige Unterschiede in den Beurteilungskategorien zu beobachten (*p*-Werte jeweils <0,05), diese waren in ihrem Ausmaß jedoch weniger bedeutend (jeweils geringe Werte für R^2^_Nagelkerke_). In den Freitextnennungen zeigte sich, dass Beanstandungen in der Beurteilungskategorie Angaben zum Unfall in jedem zweiten Fall mit unzureichenden oder fehlenden Informationen zum Unfallort einhergingen. Mängel in dieser Beurteilungskategorie wurden sowohl im niedergelassenen als auch im SAV-Bereich auf der Relevanzskala allerdings als am wenigsten gravierend eingeschätzt (Mittelwert: 4,6 bzw. 4,4). Zweifel an der Annahme eines Arbeitsunfalls besaßen dagegen die höchste Relevanz (Mittelwert: 7,0 bzw. 6,5).

### Gesamtnoten

Die Gesamtnote „sehr gut“ oder „gut“ wurde bei mehr als der Hälfte der D‑Berichte vergeben. Bei immerhin mehr als 12 % aller Fälle erhielt der D‑Bericht allerdings die Gesamteinschätzung „mangelhaft“ oder „ungenügend“. Die durchschnittliche Gesamtnote betrug 2,7 ± 1,4 im niedergelassenen und 2,5 ± 1,4 im SAV-Bereich (t-Test: *p* < 0,001, R^2^ = 0,001). Die Gesamtnote korrelierte mit einem Spearman-Koeffizienten von 0,77 (*p* < 0,001) sehr hoch mit der Anzahl pro D‑Bericht festgestellter Mängel.

In einem linearen Regressionsmodell mit einer Varianzaufklärung von 55,7 % zeigte sich ein dominierender Einfluss der Beurteilungskategorien auf die vergebene Gesamtnote, während das Alter, das Geschlecht oder andere Kontextmerkmale nur einen sehr geringen Einfluss hatten (Tab. 3). Stellte ein Peer z. B. Mängel bei der klinischen Befunddokumentation fest, so verschlechterte sich die durchschnittliche Gesamtnote eines ansonsten mangelfreien D‑Berichts von 1,587 um durchschnittlich 0,794 Noten.

**Table 3:** 

Merkmal	Merkmalsausprägung^a^	B	SE	*p*-Wert
*(Konstante)*	–	1,587	0,013	<0,001
*Alter der verletzten Person*	Minderjährig/volljährig	0,137	0,014	<0,001
*Geschlecht*	Männlich/weiblich	0,023	0,013	0,077
*Art der Beschäftigung*	Angabe/keine Angabe	0,055	0,031	0,081
*Beschäftigungszeiten*	Angaben vollständig/unvollständig	0,079	0,020	<0,001
*Unfallzeit*	Angabe/keine Angabe	0,125	0,045	<0,01
*Berichtsumfang*	Anzahl der Zeichen (zentriert)	0,000	0,000	<0,001
*Behandlungstyp*	Ambulant/stationär	0,036	0,027	0,187
*Verletzung nach VAV/SAV *[3]	Nein/ja	−0,093	0,036	<0,05
*Diagnosen*	Anzahl der Diagnosen (zentriert)	0,012	0,005	<0,05
*Bereich bzw. Einrichtungstyp*	Niedergelassen/SAV	−0,022	0,025	0,374
*Beurteilungskategorien*	Jeweils: kein Mangel/Mangel	–	–	–
Angaben zum Unfall	–	0,369	0,015	<0,001
Klinische Befunde	–	0,794	0,016	<0,001
Röntgenentscheidung	–	0,571	0,122	<0,001
Röntgenergebnis	–	0,600	0,019	<0,001
Erstdiagnose	–	0,600	0,020	<0,001
Erstversorgung	–	0,890	0,020	<0,001
Annahme Arbeitsunfall	–	1,093	0,026	<0,001
Art der Heilbehandlung	–	0,758	0,017	<0,001
Klassifikation der Verletzung	–	0,865	0,025	<0,001

Methode: Einschluss, R‑Quadrat: 0,557 (*p* < 0,001), Fälle: 20.356 (listenweiser Fallausschluss, Anm.: In diesem Modell sind nur Fälle berücksichtigt worden, in denen geröntgt wurde)

*B* Unstandardisierter Regressionskoeffizient, *SE* Standardfehler, *VAV* Verletzungsartenverfahren, *SAV* Schwerstverletzungsartenverfahren

^a^Die erstgenannte Merkmalsausprägung stellt jeweils die Referenzkategorie dar. Alle dichotomen Merkmale sind binär (0/1) kodiert

### Ebene der Leistungserbringer

Zur Erstellung der individuellen Qualitätsberichte für die Leistungserbringer wurden sowohl für den niedergelassenen als auch für den SAV-Bereich nur Leistungserbringer mit einer Mindestanzahl von 25 Bewertungen berücksichtigt. Diese wurde bei 849 D‑Arzt-Praxen und 108 SAV-D-Ärzten erreicht. Aufgrund der Mengensteuerung lagen für 816 (96,1 %) D‑Arzt-Praxen sowie für alle 108 SAV-D-Ärzte exakt 30 Bewertungen vor. Die im Oktober 2018 versandten individuellen Qualitätsberichte waren 4‑seitig und enthielten u. a. Grafiken zum eigenen Abschneiden in den 9 Beurteilungskategorien und zur durchschnittlichen Gesamtnote. Sie wiesen zudem jeweils den Mittelwert der Vergleichsgruppe sowie die Werte des jeweils besten und schlechtesten Leistungserbringers aus.

**Figure 3:**
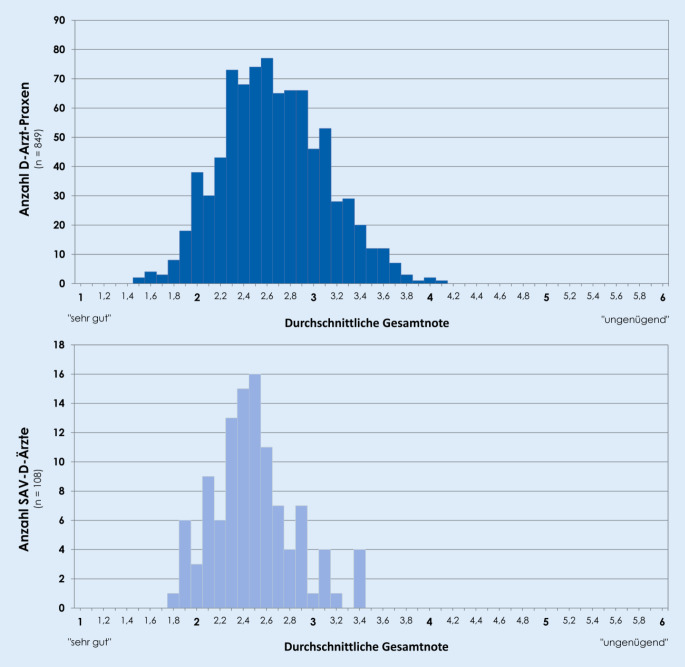

Zwischen den Leistungserbringern zeigten sich z. T. deutliche Unterschiede in der Dokumentationsqualität. In jeder Beurteilungskategorie traten sowohl herausragende (überhaupt keine festgestellten Mängel) als auch schlechte Einzelergebnisse (bis zu 80 % festgestellte Mängel) auf. Im niedergelassenen Bereich erreichten die besten D‑Arzt-Praxen eine durchschnittliche Gesamtnote von 1,5 und die schlechtesten von 4,1. Im SAV-Bereich lag das Spektrum der durchschnittlichen Gesamtnoten zwischen 1,8 und 3,4 (Abb. 3).

### Beurteilerübereinstimmung

Mit durchschnittlich 81 % für den niedergelassenen sowie 77 % für den SAV-Bereich ergaben sich gute prozentuale Beurteilerübereinstimmungen. Der für die vergebenen Gesamtnoten berechnete, durchschnittliche Finn-Koeffizient lag im niedergelassenen Bereich bei 0,49 und im SAV-Bereich bei 0,46.

### Feedback der Peers

An der anonymen Onlinebefragung beteiligten sich 59 (72,0 %) Peers, von denen alle die Checkliste als „insgesamt geeignet für die Einschätzung von D‑Berichten“ ansahen. Darüber hinaus wurde dem internetbasierten Verfahren ebenfalls eine hohe Benutzerfreundlichkeit attestiert. Einige Peers machten in Freitexten deutlich, dass sich bereits für ihre eigene Dokumentation ein unmittelbarer, praktischer Nutzen ergeben hätte. Der durchschnittliche Zeitaufwand für die Beurteilung eines D‑Berichts wurde mit 7 min angegeben. Zur Verbesserung des Verfahrens unterbreiteten einige Peers den Vorschlag, auch formale Kriterien wie Sprache, Ausdruck und Terminologie in den Fragenkatalog der Checkliste zu integrieren.

## Diskussion

### Zur Dokumentationsqualität

Mit dem vorgestellten Peer-Review-Verfahren ist erstmalig ein bundesweites Qualitätssicherungsverfahren für D‑Berichte durchgeführt worden. Dass ein Drittel der D‑Berichte keinerlei Beanstandungen aufwies, spricht insgesamt für eine gute Dokumentationsqualität.

In der Beurteilungskategorie Angaben zum Unfall, in der die meisten Mängel auftraten, ist von den Peers eine häufig zu ungenaue Schilderung des Unfallhergangs genannt worden. Für die Prüfung der Voraussetzungen eines Arbeitsunfalles seitens des UV-Trägers als Berichtsadressaten soll beurteilbar sein, ob die verletzte Person zum Zeitpunkt des Unfalls einer versicherten Tätigkeit nachging, und ob das Unfallereignis mit „hinreichender Wahrscheinlichkeit“ [5] rechtlich wesentliche Ursache für den Gesundheitserstschaden war [26]. Sprachbarrieren bei der verletzten Person können den D‑Arzt berechtigterweise an einer akkuraten Unfallanamnese hindern, sind in einem solchen Fall jedoch dann an dieser Stelle des D‑Berichts zu dokumentieren.

Ein anderer Aspekt, der fast die Hälfte aller Beanstandungen in der Beurteilungskategorie Angaben zum Unfall betraf, äußerte sich in fehlenden oder unzureichenden Angaben zu dem Unfallort. Wegen der Frage nach dem Versicherungsschutz sollte bei Unfällen im Betrieb der Betriebsteil angegeben werden (z. B. „in der Montagehalle“). Bei Wegeunfällen ist statt der unkonkreten Aussage „auf dem Weg zur Arbeit“ die Unfallstelle möglichst genau zu bezeichnen (z. B. „Kreuzung Hauptstraße/Bahnhofstraße“ oder „Gehweg nach Verlassen der Haustür“) [23]. Das zum 01.07.2018 – unabhängig von diesem Evaluationsprojekt – eingeführte neue D‑Bericht-Formular sieht mittlerweile ein separates Feld für den Unfallort vor, wodurch hier in Zukunft weniger Mängel zu erwarten sein dürften [17].

Die Überarbeitungen für die aktuell gültige D‑Bericht-Version berührten überdies auch die Beurteilungskategorie klinische Befunde mit ebenfalls relativ hohen Mängelquoten im niedergelassenen Bereich. Die Befunderhebung im gegenwärtigen D‑Bericht gliedert sich nun in die 2 Abschnitte „Beschwerden/Klagen“ und „klinische Untersuchungsbefunde“. Diese formale Änderung soll es dem berichtenden D‑Arzt strukturell erleichtern, zunächst den Beschwerdevortrag des Verletzten möglichst wortgetreu festzuhalten. Bei den klinischen Untersuchungsbefunden sind die Unfallfolgen und die hierdurch eingetretenen Funktionsstörungen ausführlich und vollständig zu beschreiben [5]. Im Fall direkter Verletzungsfolgen wie Blutergüssen, Schwellungen oder Risswunden durch Schlag, Stoß, Hieb usw. ist neben dem Sichtbefund eine exakte Lokalisationsbeschreibung erforderlich. Bei indirekten Traumata hingegen sind unbedingt die lokalen Druckpunkte anzugeben sowie ggf. typische Verletzungsfolgen und Verletzungsketten zu überprüfen. Die Befunderhebung im Bereich der Extremitäten und der Wirbelsäule ist dann vollständig, wenn auch Funktionsuntersuchungen durchgeführt und dokumentiert wurden (Stabilitätstestungen und Bewegungsausmaße) [5, 23, 26].

Die dritthäufigsten Mängel, allerdings nur den niedergelassenen Bereich betreffend, traten in der Beurteilungskategorie Art der Heilbehandlung auf. Hier zeigte sich bei der Frage, bei welchen Verletzungsmustern die Einleitung der besonderen Heilbehandlung zwingend erforderlich ist oder aber eine allgemeine Heilbehandlung als auch ausreichend angesehen werden kann, eine nicht zu vernachlässigende Unsicherheit und entsprechende Variabilität in den Entscheidungen im Hinblick auf die Heilverfahrenssteuerung.

Es wurde deutlich, dass die Anforderungen an einen vollständigen und schlüssigen D‑Bericht auch seitens der DGUV noch präziser beschrieben werden müssen. Die Landesverbände der DGUV haben angekündigt, die diesbezüglichen Erkenntnisse dieses Evaluationsprojektes auf jeden Fall in die regelmäßig stattfindenden Schulungsveranstaltungen für die D‑Ärzteschaft einfließen zu lassen. Eine ergänzende Möglichkeit böte die Ausarbeitung eines allgemeinen Leitfadens zu den Berichtsanforderungen.

Die individuellen Qualitätsberichte, welche den evaluierten D‑Ärzten zugingen, sollen eine Standortbestimmung ermöglichen und Anregungspunkte für die Weiterentwicklung ihres internen Qualitätsmanagements geben. Die Landesverbände selbst erhielten Kopien der individuellen Qualitätsberichte sowie elektronisch alle Evaluationsdaten ihres Zuständigkeitsbereiches. Um Verbesserungsmöglichkeiten aufzuzeigen, führten sie Feedbackgespräche mit jenen D‑Ärzten, welche auffällig schlechte Ergebnisse erzielt hatten. Hierbei stellte sich mitunter auch heraus, dass Dokumentationsmängel gar nicht immer nur auf die Berichtsersteller zurückzuführen waren, sondern teils durch Fehler in der zur Berichtserstellung verwendeten Software verursacht wurden.

### Zum Verfahren

Durch das vorangegangene Forschungsprojekt und die Beteiligung unterschiedlicher Experten aus D‑Ärzteschaft und UV-Verwaltung ist von der Inhaltsvalidität der Checkliste als eingesetztem Erhebungsinstrument auszugehen [8]. Mittels linearer Regressionsanalyse konnte aufgezeigt werden, dass die Beurteilungskategorien den bei weitem größten Erklärungsbeitrag für die Varianz der Gesamtnote liefern und sich die Qualität eines D‑Berichts hiermit allgemein sehr gut erfassen lässt. Andere Faktoren wie Fallschwere, soziodemografische Personenmerkmale oder der reine Berichtsumfang spielten eine eher untergeordnete Rolle. Unsere Ergebnisse deuten allerdings auch daraufhin, dass noch weitere, bisher nichtberücksichtigte Parameter für die Dokumentationsqualität von Bedeutung sein könnten. Der Hinweis einiger Peers auf rein formale Berichtskriterien (wie Ausdruck, Terminologie etc.) erscheint in diesem Zusammenhang sehr plausibel.

Naturgemäß wohnt allen Peer-Urteilen allerdings auch ein gewisser Ermessensspielraum inne, da es schwer ist, trotz standardisierter Beurteilungskriterien für alle möglichen Fallkonstellationen eine eindeutige Beurteilungsvorgabe zu formulieren. Etwas schwächere Beurteilerübereinstimmungen sind dadurch ein bekanntes Phänomen bei Peer-Reviews [9, 12]. Zudem unterliegen Wahl (u. a. [1, 9, 15, 27]) und Interpretation [10, 14, 18, 20] der Reliabilitätskoeffizienten generell immer wieder methodischen Debatten. Während die prozentualen Übereinstimmungen in den Beurteilungskategorien nach unserer Auffassung hier moderate bis gute Werte erreichten (und gegenüber dem Forschungsprojekt verbessert werden konnten [25]), lagen die Finn-Koeffizienten hinsichtlich der vergebenen Gesamtnoten nicht ganz im zufriedenstellenden Bereich, was einen limitierenden Faktor darstellt. Um ein möglichst breites Qualitätsspektrum abzubilden, wählten die Experten zur Bestimmung der Interrater-Reliabilität gleichermaßen sehr gute wie auch sehr schlechte D‑Berichte aus. Bei den schlechteren D‑Berichten zeigte sich überraschenderweise eine größere Varianz in der Notenvergabe. Da bei der Studie zur Bestimmung der Finn-Koeffizienten der Anteil als „mangelhaft“ oder „ungenügend“ anzusehender D‑Berichte gegenüber der bundesweiten Evaluation offensichtlich überrepräsentiert war, ist die „wahre“ Übereinstimmung bei den Gesamtnoten vermutlich etwas höher einzuschätzen.

Gleichwohl stellt die Verbesserung der Übereinstimmungen aus methodischer Sicht zweifellos das größte Ziel dar. Um dies zu erreichen, wurde im Projektteam bereits eine Intensivierung des Schulungskonzeptes besprochen. Da die Varianz von Urteilen natürlich auch dem Messinstrument geschuldet sein kann, scheint es angeraten, die Formulierungen von Checkliste und Manual sowie die Übungsfälle in C‑DAB, insbesondere in den Beurteilungskategorien mit geringeren Übereinstimmungen, noch einmal zu prüfen und ggf. Überarbeitungen vorzunehmen sowie weitere Übungsfälle zu entwickeln. Ebenfalls angedacht sind Präsenzveranstaltungen für die Peers, in denen die Bewertungskriterien anhand von Beispielen aus der Praxis sowohl durch einen D‑ärztlichen als auch einen UV-Vertreter aus dem Projektteam vermittelt werden. Als weitere Maßnahme wäre eine Abschlusstestung denkbar, in der Peer-Kandidaten 5 bis 10 Pilotfälle bearbeiten. Um letztlich als Peer in dem Verfahren tätig werden zu können, müssen dann bestimmte, zuvor festzulegende methodische Kriterien erfüllt sein (z. B. keine im Mittel zu stark von der Mehrheitsmeinung abweichenden Urteile) [9, 27].

Um die reale Situation für evaluierte Leistungserbringer trotz solcher methodischen Einschränkungen angemessen beschreiben zu können, wird allgemein eine Aggregation von mindestens 20 Einzelurteilen verschiedener Peers empfohlen [9, 16]. Dies war im Peer-Review der DGUV durch zumeist 30 Bewertungen gewährleistet. Überdies wurden zur Sicherung der Qualität des Verfahrens probeweise weitere Adjustierungen vorgenommen. Diese zeigten, dass das Risiko für Bevorzugungen oder Benachteiligungen von Leistungserbringern nur äußerst gering war.

Aus organisatorischer Sicht ist zu betonen, dass die prospektive Datenerhebung hier auf der Grundlage von elektronisch verfügbaren Routinedaten gänzlich papierlos und standortunabhängig erfolgen konnte und nach Rückmeldung aller Beteiligten eine hohe Praktikabilität aufwies. Dies ermöglichte überhaupt erst eine systematische Evaluation mit derart großen Fallzahlen.

Ziel der DGUV ist es, die Qualität der D‑Berichte noch weiter zu verbessern. Deren Gremien haben deshalb den Beschluss gefasst, das Peer-Review in Zukunft fortzuführen und dabei neben den bisherigen auch weitere Gruppen von Leistungserbringern einzubeziehen. Ein geplanter Längsschnittvergleich mit bereits evaluierten D‑Ärzten soll die relevante Frage beantworten, ob das vorgestellte Verfahren wirklich zu messbaren Verbesserungen der Dokumentationsqualität beitragen konnte.

## Fazit für die Praxis

- Erstmals wurde die Qualität von D‑Berichten bundesweit systematisch erfasst. Die Deutsche Gesetzliche Unfallversicherung (DGUV) ist hiermit einer satzungsgemäßen Aufgabe nachgekommen (Sicherung der Prozessqualität).
- Die Dokumentationsqualität war insgesamt gut. Zwischen den einzelnen Berichtserstellern bestehen z. T. jedoch große Unterschiede.
- Evaluierte D‑Ärzte erhielten individuelle Qualitätsberichte, um ihre eigene Berichtsqualität im Sinne eines Benchmarkings einschätzen zu können.
- Zur weiteren Verbesserung der Berichtsqualität sollten die DGUV und ihre Landesverbände die Anforderungen an die Berichtserstellung z. B. im Rahmen der D‑ärztlichen Schulungsveranstaltungen noch klarer vermitteln.
- Das elektronische Evaluationsverfahren konnte sich bewähren, weshalb Wiederholungen unter Beteiligung weiterer D‑Arzt-Gruppen geplant sind.

## Footnotes

1 Aus Gründen der besseren Lesbarkeit wird auf die gleichzeitige Verwendung *männlicher, weiblicher und neutraler* Sprachformen verzichtet. Sämtliche Personenbezeichnungen gelten gleichwohl für *alle Geschlechter*.
